# How can the impact of national recommendations for physical activity be increased? Experiences from Germany

**DOI:** 10.1186/s12961-018-0396-8

**Published:** 2018-12-14

**Authors:** Alfred Rütten, Karim Abu-Omar, Sven Messing, Mayra Weege, Klaus Pfeifer, Wolfgang Geidl, Verena Hartung

**Affiliations:** Department of Sport Science and Sport, Friedrich-Alexander-University Erlangen-Nürnberg, Gebbertstraße 123b, 91058 Erlangen, Germany

**Keywords:** Public health, Physical activity promotion, Recommendations, Implementation, Impact, Policy, Politics, Multiple streams

## Abstract

**Background:**

Clearly stated national recommendations for physical activity (PA) are available in many nations. Yet, their impact on national level policy-making might be considered modest at best. This paper analyses the approach selected to curtail this problem in Germany.

**Main text:**

Academics, government institutions and relevant stakeholders worked in collaboration to produce a series of national recommendations for PA and PA promotion published in 2016. Since their publication, these recommendations have achieved a measurable political impact through a systematic approach focused on dissemination and implementation strategies. For example, the German Conference of Health Ministers has acknowledged the national recommendations, endorsed their dissemination and proposed that they are included in measures within the Federal Prevention Act.

**Conclusion:**

Since their publication, the national recommendations have yielded satisfactory political impact. The contributing aspects might partially be explained by the Multiple Streams Approach.

## Background

The prevalence of unmet physical activity (PA) recommendations in both child and adult populations [1] and the related increase in major non-communicable diseases [2] warrants steadfast efforts in PA promotion. Global recommendations concerning PA have been established since 2010 [3] and are regarded as an important stepping-stone in PA promotion advancements. There are increasing calls for the creation of national recommendations, positing their function as a key element in aligning relevant actors to promote the PA agenda within countries and to increase the PA promotion drive [4–6].

Across the world, a large number of nations have responded to these calls and endeavoured to formulate national recommendations. According to Leone and Pesce [7], there are two main types of recommendations regarding PA. The first type derives from evidence-based clinically guided frameworks and concerns duration, intensity and volume of PA. These recommendations focus on health-enhancing levels of PA, providing specific guidance on PA amounts and modalities through different fitness levels and age groups. Globally, the United States [8] and Canada [9] figure as examples of countries publishing such recommendations. In Europe, 21 out of 37 analysed countries [10], including the United Kingdom [11], Austria [12] and Switzerland [13], publish their own recommendations.

The second type of document, referred to as PA promotion recommendations, arises from a different perspective. It attends more often to stakeholders such as practitioners, professionals and organisations involved with prevention and health promotion, as well as political decision-makers and institutions. Recommendations for PA promotion target evidence-based interventions in specific settings, concentrating on daily living. Their intention is not to define the amount and frequency of PA practice, but rather to inform policy actions on how to promote health-enhancing PA [7]. Scientists have prompted the development of national recommendations concerning evidence-based interventions and policies [14]. In a global context, WHO has addressed this matter through the creation of PA promotion recommendations [15].

However, a lack of systematic connection between PA recommendations and PA promotion efforts still remains. A Canadian study has shown that solely publishing PA recommendations has a low impact on population reach [16]; the unprompted recall of such recommendations among the population was reported to be as low as 4%, exposing a need for more coordinated strategies regarding PA promotion. Nevertheless, most of the national recommendations published thus far do not include recommendations on PA promotion. Such recommendations could provide guidance for organisations on how to promote PA and improve PA behaviour among the population. The limited emphasis on recommendations for PA promotion might impact PA promotion practice, PA policies and, ultimately, PA prevalence.

Nevertheless, endeavours to inform policy action regarding PA promotion have been gradually advancing. Observing that dissemination-based strategies had proved inefficient, Canadian efforts have moved towards targeting implementation and behaviour change in order to impact public health [17]. They strived for innovative approaches encompassing changes in policies and services. In the United States and the United Kingdom (e.g. [18]), national agencies have published scientific documents on specific aspects of PA promotion. Both the European Union and WHO have encompassed the expansion of efforts for PA promotion in their political agenda [4, 5, 19, 20]. Furthermore, strategies for holding nations accountable [5, 21] and frameworks assessing national PA promotion efforts [22] have been developed.

### Context of physical activity promotion in Germany

In view of the global context, Germany’s PA levels and PA promotion reality do not differ greatly from other developed nations. A nationally representative study of children and adolescents aged between 3 and 17 years indicated that only 29% of boys and 22% of girls are sufficiently physically active [23]. Additionally, only one out of five adults fulfils the WHO recommendations [24]. However, Germany displays its own particularities in dealing with PA and PA promotion among its population. A large proportion of the German population is active in sports clubs – in 2017, more than 27 million people belonged to a sports club [25]. Data from Eurobarometer states that the proportion of people exercising is higher [26] and that cycling is more common than in most other European countries [27]. In contrast, the amount of walking is close to the average of EU member states [27].

The German political system divides PA promotion responsibilities among several stakeholders at different levels. Rather than merely sharing the burden, such a structure might affect efficiency and could impair decisive action. While the Federal Ministry of Health, for example, is responsible for health promotion initiatives and the promotion of everyday PA, the Federal Ministry of the Interior is responsible for elite sports. Germany is a federal republic composed of 16 states, where each state assumes a substantial role in PA promotion due to the system configuration. The main responsibility for PA promotion in school settings lies with the Education Ministries of the federal states. Organisations such as sports clubs and health insurance companies take on responsibility in the development of PA promotion at local, regional and federal levels. Based on this, experts have expressed the need for more sustainable structures, describing the current efforts in Germany as fragmented [28].

At the national level, PA promotion has often been addressed in relation to nutrition and developing strategies and policies for healthy living. The Federal Ministry of Health and the Federal Ministry of Nutrition cooperate, for example, in the initiative IN FORM [29], which targets improvements in PA and nutrition behaviour. Based on a national action plan, IN FORM has marked a starting point for increasing efforts in the field on a national level since 2008. Actions as these have increased over the past years in Germany, aiming to strengthen the preventive aspect of a healthcare system that has traditionally been more curative oriented [30]. As a result, the Federal Prevention Act was adopted in 2015 [46].

### What this study contributes

This article intends to start a debate on how to increase the political impact of national PA recommendations. In order to do so, the development, dissemination and implementation of the German recommendations are described. A coherent process aimed at evenly encompassing relevant stakeholders was set in motion based on an approach which systematically links (1) PA recommendations and (2) recommendations for PA promotion. It might also assist other nations in the process of developing and implementing their own national recommendations.

Currently available literature focuses on the development and content of recommendations [31–34]. Communication and implementation strategies are presented as a next step [14]; however, this part of the process is seldom described or analysed. Therefore, the present paper not only describes the development of PA and PA promotion recommendations (phase 1), but also outlines their dissemination and implementation supported by the engagement of stakeholders (phase 2) as well as political impact (phase 3).

## Main text

### Phase 1: development of recommendations

A comprehensive description of the development of the German recommendations for PA and PA promotion – from origin to publication – is provided. It details (1) the process, evolving from a topic on the political agenda to a feasible project with proper financial and human resources, (2) the devised structure to ensure that the recommendations reach political impact and target populations, and (3) the methodology, which accredited the recommendations with scientific credibility.

#### Political process

Experts’ call for developing ‘recommendations for PA promotion’ in Germany can be traced back to a colloquium organised by the German Conference of Sports Ministers a few years ago. Several political institutions have since joined in the request. In 2015, the German Conference of Sports Ministers and the German Conference of Health Ministers agreed to jointly request the development of PA recommendations. In parallel, a consulting board previously established to advise the Federal Health Ministry on PA matters (called the Physical Activity Promotion in Daily Living working group) commenced developing the National Recommendations for Physical Activity and Physical Activity Promotion. This consulting board was initiated by scientists in the field of sport science in Germany and is comprised of relevant stakeholders from both health and sports sectors, as well as members from the academic community with expertise in the field. Once the academics confirmed the possibility to produce the scientific evidence and the Ministry of Health succeeded in guaranteeing allocation of proper funds, the development of national recommendations was set in motion. Several academics of sport science and sport medicine,^1^ supported by an international scientific advisory board,^2^ compiled the scientific evidence in a project that took place between July 2014 and December 2015. Based on the collected knowledge, the National Recommendations for Physical Activity and Physical Activity Promotion were published in September 2016 [35].

Reflecting upon the political aspect, several factors strengthened the process, yet certain key elements most prominently contributed to its success. Firstly, the creation of a scientific consensus in the field of PA and PA promotion in Germany by engaging renowned academics increased the credibility of the recommendations. It also guided the development process as a scientific endeavour rather than a political one. Secondly, the decision to write recommendations that explicitly target stakeholders later facilitated having them work as multipliers to target groups and implementers of PA promotion measures. This granted a connection to real-world conditions. Finally, the political and financial support provided by the Federal Ministry of Health provided the favourable circumstances needed to reach the proposed goals. The combination of theoretical strength, practical soundness and auspicious political timing seems to have aided the fruition of the project.

#### Selection of the content and structure of the recommendations

It is important to highlight the strong connection between recommendations for PA and PA promotion in the resulting document. Both aspects hold equivalent weight, length and level of detail. The PA recommendations comprise chapters on children and adolescents, adults, older adults and adults with chronic diseases. The recommendations for PA promotion encompass the same chapters, plus an additional one regarding the general population (Table 1).

**Table 1:** Structure of Germany’s national recommendations for physical activity (PA) and PA promotion

	Recommendations for PA	Recommendations for PA promotion
Chapters	Children and adolescents	Children and adolescents
	Adults	Adults
	Older adults	Older adults
	Adults with a chronic disease	Adults with a chronic disease
		General population

Beyond the target group, PA promotion recommendation chapters are organised by settings. Concerning children and adolescents, for example, recommendations target interventions in child day-care, school and household settings. This structuring differs from other PA promotion recommendations, which are framed by intervention type [36] or by a mix of target group, setting and intervention type [15]. The decision to structure the recommendations by setting was made in favour of a stakeholder-focused approach, intending to support both dissemination among stakeholders and uptake of recommendations by stakeholders in each setting. Additionally, it is essential to highlight that the national recommendations conform to the Federal Prevention Act, which greatly emphasises the importance of disease prevention in different settings. This ensures that the PA recommendations meet requirements for funding stipulated by the Federal Prevention Act [46].

#### Scientific methods pursued to draft the recommendations

The following section represents a brief account of the methodological process performed. PA recommendations were developed through a three-stage process conducted by the involved academics. PA recommendations followed the protocol described by Geidl and Pfeifer in 2016 [37]. The three-stage process was as follows:

1. Systematic literature search for current PA recommendations. Additionally, criteria for assessing the quality of these recommendations were developed in an expert consultation.
2. Evaluation of PA recommendations based on quality criteria. High-quality recommendations were identified and their content analysed.
3. Synthesis of content analysis and formulation ensuing from German PA recommendations.

The recommendations for PA promotion resulted from three comprehensive literature reviews on the topics of efficacy/effectiveness of PA interventions [38], cost-effectiveness of PA interventions [39, 40] and quality criteria for their implementation [41]. Early on, it was decided that the main goal was to establish a firm evidence base for PA promotion in order to ensure political impact. The goal was to provide a variety of interventions from which stakeholders could chose the one(s) that best applied to their needs. Therefore, the following questions were asked:

- Which PA interventions have demonstrated efficacy (internal validity) and effectiveness (external validity)?
- Which PA interventions are cost-effective?
- Which PA interventions have the potential to reduce existing health inequalities?
- Which good practice criteria are available to guide the implementation of PA interventions?

In order to answer these questions, the following reviews were conducted:

1. Systematic review of reviews on the efficacy and effectiveness of interventions [38], which ultimately identified 213 reviews that dealt with the topic of PA promotion. Six electronic databases were searched in the process. Included reviews contained results of empirical single studies of interventions that either focused on PA promotion or reduction of physical inactivity, reported results on the efficacy/effectiveness of those studies, and were published in English or German language. The process of screening and including the reviews was conducted by two independent reviewers. Additionally, one independent researcher assessed the methodological quality of all included reviews by using the AGREE instrument [42]. This review was also later used to address the issue of health inequalities.
2. Systematic review on the cost-effectiveness of interventions [39, 40]. This review of reviews identified 18 reviews that dealt with the topic of cost-effectiveness of interventions for PA promotion. Ten international databases were searched in the process of identifying the reviews. The included reviews modelled or summarised a health economic evaluation of an intervention for PA promotion, documented their search strategy and inclusion and exclusion criteria, targeted healthy adults, and were published in English or German language. The screening process was conducted by two independent reviewers. A third reviewer utilised the National Collaborating Centre for Methods and Tools instrument [43] to assess the methodological quality of all included reviews.
3. Review of interventions quality criteria [41]. This review followed the methodology proposed by Grant and Booth [44]. The search was conducted in two scientific databases; grey literature from governmental or non-governmental organisations was identified through Google Web Search. Documents were included if they contained quality criteria on the conceptualisation, implementation and evaluation of interventions for PA promotion, and were published in English or German language. The process identified 24 documents that were used to extract relevant quality criteria.

The yielded results provided the evidence needed to draft the recommendations for PA promotion. A more detailed description of the methodology employed to formulate the German recommendations for PA promotion is published elsewhere [45].

### Phase 2: Dissemination and engagement of stakeholders

At the end of phase 1, the national recommendations were published [35]. In phase 2, we describe the (1) process and (2) outcomes of engagement of stakeholders such as practitioners, professionals and organisations. This aimed at effectively disseminating and implementing the recommendations. Considering that the recommendations in their entirety lend as much importance to guidance on PA as guidance on PA promotion, the dissemination phase followed the same logic. In order to maximise the impact of recommendations, efforts focused on engaging stakeholders in a dialogue in order to raise awareness about the recommendations, facilitating their uptake and implementation, ultimately transforming stakeholders in multipliers of the recommendations. Stakeholders were, simultaneously, target group and active participants in this process.

#### Process of stakeholder engagement

**Fig. 1:**
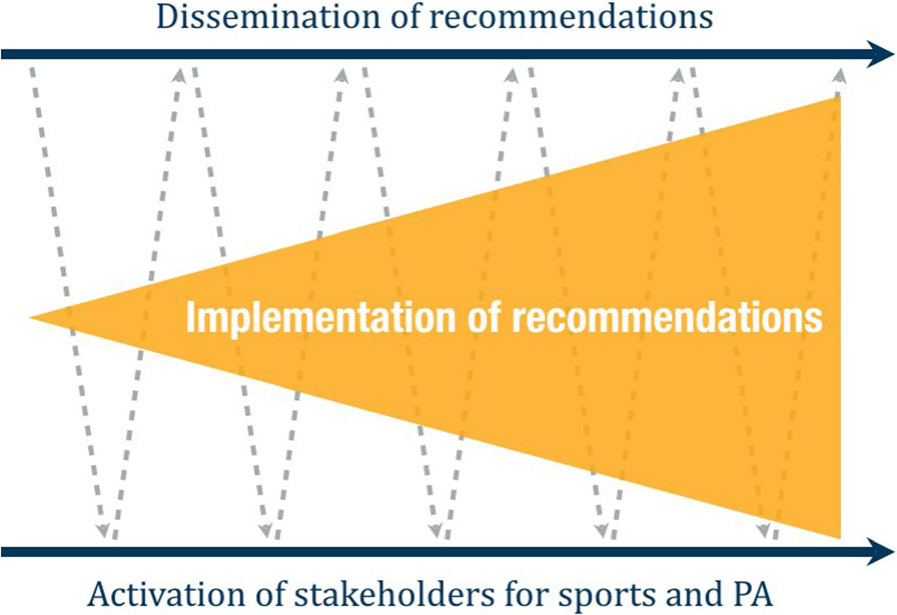
Model for the implementation of Germany’s national recommendations for physical activity and physical activity promotion

The second phase of the project was again led by the national recommendations’ authors and funded by the Federal Ministry of Health. Given the scientific nature of the recommendations published at the end of phase 1, phase 2 targeted increasing impact through the development of strategies and materials for broader dissemination of the recommendations among stakeholders. The academics’ understanding assumed both recommendation dissemination and stakeholders’ engagement as fundamental complementary components for effective implementation of the recommendations in the public health practice (Fig. 1). It also presumed them to work interdependently, where the stakeholders’ engagement reinforced the dissemination of recommendations and vice versa.

Supported by the Ministry of Health, academics organised two high-level workshops with 60–80 participants in 2017 in order to engage stakeholders. The workshops served three purposes, namely (1) introducing the recommendations to stakeholders; (2) engaging stakeholders in the process of drafting advertising materials, which allowed the recommendations broader reach among multipliers; and (3) developing initial strategies for dissemination and implementation of the recommendations. For this, participants were divided into five groups (one for each target group). During the time between the two workshops, stakeholders were asked to confer within their groups in order to advance their efforts.

When selecting stakeholders to take part in the workshops, a sampling matrix was used to ensure the involvement of representatives of all relevant settings. This matrix was structured by target group and setting, in agreement with the PA promotion recommendations. The matrix comprised general settings, relevant for all target groups (sports, healthcare, community) and specific settings (e.g. school for children/adolescents, worksite for adults and nursing homes for older adults). For each matrix cell, two different types of stakeholder were contacted, specifically stakeholders with close proximity to the target groups (e.g. coach of a local sport club) and representatives with decision-making power from relevant stakeholder organisations (e.g. member of the umbrella organisation of all sport clubs at national level). This was done to ensure that the resulting strategies and materials reflected the needs of stakeholders at all levels. Additionally, a high-level representative from the Ministry of Health also joined. Approximately 65% of contacted stakeholders agreed to take part in the high-level workshops.

#### Outcomes of stakeholder engagement

One of the main outcomes of the stakeholder engagement process was a reader-friendly version of the recommendations. Such a document was necessary due to the scientific density of the published national recommendations. It was developed in collaboration with a marketing agency and revised by the participating organisations. In order to create the final version, the stakeholders’ suggestions for improvement were considered and its intelligibility was increased. The resulting document is currently in the process of being published by the Federal Centre for Health Education, which shall both print it and start its large-scale distribution to stakeholders.

Stakeholders were asked to cooperate in five groups (one for each target group). Each group was asked to develop strategies (i.e. which stakeholders to target, as well as ideas and possibilities for reaching them) and advertising materials (i.e. brochures, flyers, videos) for the dissemination of recommendations towards relevant groups of stakeholders. The resulting strategies and materials addressed leaders of childcare facilities and schools (children and adolescents), companies and universities (adults), senior organisations (older adults), doctors and medical associations (adults with chronic diseases), and political decision-makers as well as the administration (general population). The different groups deemed these as the most important target stakeholders.

Examples of strategies devised by the general population group included (1) informing decision-makers and administrative staff about the recommendations, (2) motivating these stakeholders to implement the recommendations, (3) improving intersectoral collaboration with other relevant sectors for PA promotion, and (4) increasing the participation of different stakeholders in PA promotion efforts. Examples of ideas for materials devised by the general population group included the creation of an information package about the national recommendations, including brochures and flyers, an information package about examples of good practice, and a tool package that integrates setting specific materials developed by the other groups.

### Phase 3: political impact

One of the aims of phases 1 and 2 was the generation of impact in the field of PA promotion through national recommendations. In order to open a debate on political impact, (1) current developments are discussed and (2) a possible theoretical approach to explain them is described.

#### Examples of political impact in Germany

The latest developments suggest that national recommendations are generating political impact and supporting the development of evidence-based policies. Relevant instances have taken place in the comparatively limited time elapsed since publishing the recommendations. For example, (1) in 2017, the German Conference of Health Ministers passed a resolution acknowledging the national recommendations, endorsing their dissemination and proposing that they are included in measures within the Federal Prevention Act. The Federal Prevention Act aims to strengthen health promotion and prevention in Germany, especially by supporting activities of the statutory health insurances in settings such as childcare, school, community, workplace and care facilities, with at least €300 million per year [46]. As a consequence, the endorsement of the recommendations by the Conference of Health Ministers might facilitate the access to funding and thus the implementation of measures for PA promotion. (2) The German Medical Assembly approved a resolution advising physicians to inform themselves about the recommendations for PA and PA promotion. The resolution emphasises the endorsement for the implementation of PA counselling in practices, clinics and administrations as an effective measure of PA promotion [47]. Although the resolution as such is not compulsory to physicians, it is now used by stakeholders to lobby for the implementation of exercise prescription systems. Such systems are currently being piloted in Germany; however, they have not yet been widely adopted. (3) The guidance document of the statutory health insurances now refers to the national recommendations for PA and PA promotion [48]. This guidance document is the basis for all funding activities of the statutory health insurances; measures that do not meet the criteria stated in this document cannot be supported by any statutory health insurance. It is expected, henceforth, that further measures targeting the implementation of the national recommendations will be undertaken and thus increase their impact. (4) Finally, the Federal Ministry of Health has now issued a call for project proposals [49]. The call seeks projects that explicitly implement the German PA recommendations at the local level. This call should increase research activities in the field of PA promotion in Germany while at the same time strengthening the role of the Federal Ministry of Health in this area.

#### Understanding the political impact of the recommendations

Opening a debate on the political impact of national guidelines is crucial to advancements in the field. Attempting to explain the entailments of the expedited results obtained serves the purpose of starting a discussion and maybe assisting nations in developing and implementing their own version of such a process. In order to facilitate assimilation, we have employed the Multiple Streams Approach (MSA) to elucidate the proposed reasoning. Granted that analyses provided are of a tentative nature, inferences are believed to aid in reproducing attained outcomes.

The MSA was developed in the United States in 1984 to explain agenda-setting [50]. Originally based on case studies in public health and transportation policy, it is currently used in Europe to explain political processes as well as different policy domains [51]. In a nutshell, it conjectures that the political process encompasses three streams that flow independently, namely (1) the problem stream, which contains specific issues perceived as problematic and calling for a solution, (2) the policy stream, which addresses the development of ideas and possible policies presently available to address the stated problem, and (3) the politics stream, which comprises operating actors – political parties, institutions and interest groups. A policy entrepreneur can coalesce the three streams and introduce a specific topic into the political agenda when a window of opportunity opens.

In the present case, the coupling of streams might have meant that PA promotion was included in the political agenda as a stand-alone topic (differently from past scenarios, when it circulated paired with nutrition or other health behaviours). Applying the MSA frames our case as follows:

1. Problem stream: Low prevalence of PA is perceived as a problem since regular PA has been linked to lower mortality rates [52, 53], lower risk for major non-communicable diseases [2], as well as other positive effects on health. A study conducted concurrently to the development of national recommendations showed the health-economic costs of physical inactivity in Germany to be approximately €14.5 billion or 4.8% of the national healthcare costs [54]. Researchers have described physical inactivity as a chronic policy problem [55] and institutions such as WHO, the European Commission and the Council of the European Union have also identified it as an issue [4–6]. This has increasingly built pressure on political systems, calling for actions to promote PA.
2. Policy stream: Several existing health-promoting policies were used to solve the problem of low PA prevalence and support PA promotion. In Germany, the following were of outstanding importance: (1) the Social Insurance Code, which mandated statutory health insurances to offer services for health promotion and prevention in settings; (2) the Federal Prevention Act, which created structures such as coordination bodies for the implementation of health promoting policies; and (3) policies outside of the health sector that are relevant for PA promotion such as sports facility development on the local level. All these policies could be exploited to increase the political impact of the recommendations for PA and PA promotion, especially in the dissemination and implementation phase.
3. Politics stream: In phase 1, confirmation of support for the development of recommendations from existing bodies such as the Conference of Health Ministers, the Conference of Sport Ministers and the Federal Ministry of Health consulting board was imperative. In phase 2, incorporating important interest groups from all relevant political sectors into the process was crucial to increase the acceptance of the recommendations and to motivate each group to actively support implementation and dissemination. In phase 3, there are indications that the politics stream actors uphold their support. All these instances provided important political support, thus making it possible for the recommendations to achieve political impact.
4. Policy entrepreneurship: The array of actors who played an important role is one of the perceived key aspects for the success of this case. Firstly, the expert staff of the Ministry of Health, who pioneered in making PA promotion a stand-alone topic on the national agenda. Secondly, the leading academics in the field, who developed the recommendations and organised the process for dissemination and stakeholder engagement. Thirdly, the Federal Minister of Sport and the Federal Minister of Health, who displayed crucial political endorsement by writing a personal acknowledgement address.
5. Window of opportunity: The expert staff of the Ministry of Health and the leading academics opened the ‘window of opportunity’. They acted as policy entrepreneurs and managed to couple the three streams. The national recommendations for PA and PA promotion were (a) perceived to deal with a relevant problem (problem stream), (b) in line with relevant health-promoting policies (policy stream) and (c) supported from relevant institutions and interest groups (politics stream). Thus, the time was ripe for the development of national recommendations for PA and PA promotion in Germany.

It is important to reiterate that the presented interpretation through MSA is a tentative approach to understanding the results obtained, contributing to the discussion on the political impact of national recommendations. Beyond any conclusions, it aims at enriching the debate by offering distinctive perspectives derived from experience.

## Discussion

### Study findings

A distinct call for advancements in the field prompted the formulation of national recommendations for PA and PA promotion in Germany. The Federal Ministry of Health supported the process thoroughly by providing human and financial resources, as well as political endorsement. The resulting recommendations strived to generate impact by adopting a systematic approach. This approach aimed at guaranteeing that interdependencies between PA recommendations and PA promotion recommendations would be addressed, ensuing a scientifically rigorous developmental process, which granted credibility to the resulting documents. It also gave dissemination and implementation a greater role. The engagement of key stakeholders in designing strategies and materials ensured connection to real-world settings and favoured broader reach. The recommendations have thus far yielded highly promising political outcomes. The specific reasoning as to how our methods are linked to such an impact can be explained by the MSA. At the same time, the approach employed in Germany raises a number of more conceptual issues.

### Distilling what other nations might and might not learn from Germany

For other nations planning to increase the political impact of their PA promotion efforts, we suggest (1) the development of national recommendations, which aids PA pushes onto the political agenda and drives PA promotion to gain momentum. (2) Making the development of PA recommendations a collaborative process, including individuals with expertise in the field. Engaging a group of academics, for example, lends credibility to the resulting document and most likely improves the quality and appropriateness of the information yielded. (3) Linking PA recommendations and PA promotion recommendations, combining them in one document and giving equivalent weight to both, thus curtailing the gap between guidance and practice and facilitating action-taking by key organisations. (4) In the dissemination phase, using an intersectoral and participatory process to engage stakeholders. Such an approach increases the likelihood that the created materials and strategies will indeed fit to the needs of all stakeholders by involving them in all activities. Finally, (5) guaranteeing backing by a federal agency will greatly impact the probability of success. This impact is even higher when such an organisation also has the possibility to fund the process. Political support is vital, especially when considering impacting on policies. The financial aspect brings logistic ease. Thus, although not imperative, the possibility of obtaining both political and financial support from the same source should be considered when aiming at a successful development and implementation of national recommendations.

It is, however, important to clarify that the generalisability of our approach is quite limited. MSA itself states that an element of ambiguity is indeed involved – the coupling of streams falls beyond the realm of control of any involved actor, and this implies that the sheer replication of our efforts might not result in guaranteed success. Researchers in other nations might follow our suggestions and still fall short of their objectives. This can be partly explained by the high degree of influence that the national context exerts on PA promotion. From political parties to governance systems, each nation represents a unique case study on how to enhance efforts for PA promotion. This understanding ought to serve as incentive for researching the vast, albeit complex, topic of how to increase the impact of national PA recommendations.

### Researching ways to increase the impact of PA recommendations

Eventually, different nations seem to take differing approaches on how to increase the impact of PA recommendations. In the United Kingdom and the United States, these efforts have resulted in a collection of corollary documents (e.g. [18, 56]) on how best to increase PA. In Canada, tools to increase the public health impact of the recommendations now include interdisciplinary collaboration, policy change and refocused service provision [17]. Through these, increases in the uptake of the recommendations by organisations have been made [17] and evaluated [57]. Researchers in other nations have developed annual report cards on PA policies (e.g. [58, 59]), and WHO Europe has begun to publish bi-annual country fact sheets on PA prevalence and promotion efforts for Europe [21]. Both set out to make existing PA policies in different states or nations publicly comparable. Ultimately, behind those different approaches to influence policy development for PA promotion lies a research agenda for scientists on how to best influence policy development and measure its potential impact.

## Conclusion

Further efforts by academics and stakeholders targeted at enhancing the impact of PA recommendations are warranted. Stronger connections between PA recommendations and PA promotion recommendations on a national level are an essential first step down that path. Collaboration within nations and among sectors provide the opportunity to increase the influence of recommendations and generate a higher overall impact of public health.

## Abbreviations

MSA: Multiple Streams Approach
PA: physical activity

## Footnotes

1 The academic group consisted of Alfred Rütten, Klaus Pfeifer, Winfried Banzer, Christine Graf, Ulrike Ungerer-Röhrich, Klaus Völker and Lutz Vogt, as well as of several research associates and research assistants (see authors of the national recommendations).

2 The international scientific advisory board consisted of Klaus Bös, Sonja Kahlmeier and Willem van Mechelen.
